# Oxaliplatin-based versus irinotecan-based hyperthermic intraperitoneal chemotherapy (HIPEC) in patients with peritoneal metastasis from appendiceal and colorectal cancer: a retrospective analysis

**DOI:** 10.1186/1471-2407-14-807

**Published:** 2014-11-04

**Authors:** Gabriel Glockzin, Michael Gerken, Sven A Lang, Monika Klinkhammer-Schalke, Pompiliu Piso, Hans J Schlitt

**Affiliations:** Department of Surgery, University Hospital Regensburg, 93042 Regensburg, Germany; Tumor Center Regensburg e. V., University Regensburg, Regensburg, Germany; Department of Surgery, Hospital of the Order of St. John of God, Regensburg, Germany

**Keywords:** Peritoneal carcinomatosis, HIPEC, Irinotecan, Oxaliplatin, Morbidity, Survival

## Abstract

**Background:**

Cytoreductive surgery (CRS) and hyperthermic intraperitoneal chemotherapy (HIPEC) provide an effective treatment option for selected patients with colorectal peritoneal metastasis with encouraging survival results. Many different drug combinations and HIPEC regimens including bidirectional, i.e. synchronous intravenous and intraperitoneal, drug application have been used. However, there is still no standardization of the HIPEC regimen.

**Methods:**

Between 05/2007 and 04/2010 190 patients underwent CRS and HIPEC at the University Hospital Regensburg. Thirty-two patients with peritoneal metastasis arising from colorectal or appendiceal cancer underwent complete macroscopic cytoreduction (CC-0/1) and bidirectional HIPEC and completed at least 3-year follow-up. Twenty patients received oxaliplatin-based (OX) and twelve patients received irinotecan-based HIPEC (IRI). Group-specific perioperative morbidity and 3-year survival has been determined.

**Results:**

The grade 3/4 morbidity rate according to CTCAE v4 was 35.0% in the OX group vs. 33.3% in the IRI group (p = 1.000). There was no perioperative mortality in both groups. Median survival was 26.8 months (95% CI 15.7-33.1 months) in the IRI group and has not yet been reached in the OX group during a median follow-up of 39.4 months. Three-year survival rates were 65.0% in the OX group vs. 41.7% in the IRI group (p = 0.295).

**Conclusions:**

The morbidity and toxicity rates of bidirectional irinotecan-based and oxaliplatin-based HIPEC are comparable. Nevertheless, in the absence of contraindications oxaliplatin-based HIPEC might be preferred due to the positive trend regarding 3-year and median survival.

## Background

The combined treatment concept consisting of cytoreductive surgery (CRS) and hyperthermic intraperitoneal chemotherapy (HIPEC) performed in specialized centers has shown to be a safe and efficient additive therapeutic option for selected patients with colorectal peritoneal metastasis [1–3]. One prospective randomized controlled phase III trial and several prospective and retrospective reports provide evidence for improved long-term survival for CRS and HIPEC as an integrative part of an interdisciplinary treatment regimen [4–9]. In the Dutch RCT the median survival of patients who underwent CRS and HIPEC was 22 months vs. 12.6 months in the control group with systemic chemotherapy only. In the subgroup analysis of patients after complete macroscopic cytoreduction (CC-0/1) median survival increased to 42.9 months [8, 9]. As in most other reported studies and series a mitomycin C (MMC)-based HIPEC regimen has been used for peritoneal perfusion. Based on the results of modern systemic polychemotherapy regimens such as FOLFOX or FOLFIRI for patients with metastatic colorectal cancer oxaliplatin and irinotecan have also been used for peritoneal perfusion. Data first published from the French groups suggest that oxaliplatin-based HIPEC after complete macroscopic cytoreduction may further improve survival of patients with colorectal peritoneal metastasis [10, 11]. The addition of intraperitoneal irinotecan to the bidirectional oxaliplatin-based HIPEC regimen did not lead to improved overall or relapse-free survival [12]. Nevertheless, as irinotecan is considered to be the second most effective agent for the treatment of patients with colorectal cancer [13, 14], bidirectional irinotecan-based HIPEC might be a promising alternative treatment regimen for patients with disease progression or intolerable adverse events under oxaliplatin-based chemotherapy as well as patients with good response under previous systemic chemotherapy with irinotecan. However, conclusive data from randomized controlled trials is still missing and numerous different HIPEC regimens are used for treatment of colorectal peritoneal metastasis [15]. Cytostatic agents, drug dosage and duration of perfusion are still a matter of debate.

In the present study we retrospectively analyzed morbidity, mortality and 3-year survival of thirty-two patients with peritoneal metastasis arising from colorectal or appendiceal cancer who received either bidirectional oxaliplatin-based or irinotecan-based HIPEC after complete macroscopic cytoreduction.

## Methods

Between May 2007 and April 2010 190 patients underwent cytoreductive surgery (CRS) and hyperthermic intraperitoneal chemotherapy (HIPEC) for various peritoneal surface malignancies at the University Hospital Regensburg. Thirty-two patients with synchronous or metachronous peritoneal metastasis arising from colorectal or appendiceal cancer received bidirectional HIPEC after complete macroscopic cytoreduction (CC-0/1). Twenty patients received oxaliplatin-based HIPEC and twelve patients received irinotecan-based HIPEC. All patients had histologically proven peritoneal carcinomatosis arising from colorectal or appendiceal adenocarcinoma. Patients with disseminated peritoneal adenomucinosis (DPAM) or peritoneal mucinous carcinomatosis of intermediate features (PMCA-I) as well as patients with incomplete macroscopic cytoreduction (CC-2 or CC-3) were excluded from the present study.

Data has been analyzed retrospectively. The retrospective analysis from a database without the use of patients’ personal data was exempted from approval by the Ethics Committee at the Regensburg University. Nevertheless, CRS and HIPEC are recommended for selected patients by the German S3-guideline for the treatment of colorectal cancer [16]. Moreover, the bidirectional oxaliplatin-based HIPEC regimen has been approved by the ethic committee in the context of our prospective multicenter phase II COMBATAC trial (ClinicalTrials.gov Identifier: NCT01540344) [17] and is recommended as one of the standard HIPEC protocols for patients with colorectal peritoneal metastasis by the German Peritoneal Surface Malignancy Group. The individual reasons for the replacement of oxaliplatin by irinotecan in the IRI group are summarized in Table 1. The safety of intraperitoneal application of irinotecan has been proven in several published studies [12, 18, 19]. However, due to the lack of consistent data there are still no national and/or international standards for HIPEC regimens in patients with colorectal peritoneal metastasis [15].

**Table 1:** **Characteristics of patients with irinotecan-based HIPEC**

Patient	Previous syst. CTx	Previous syst. OX	Recurrent PM	Rationale for irinotecan-based HIPEC
Patient 1	yes	yes	yes	Oxaliplatin-associated peripheral neuropathy
Patient 2	yes	yes	no	2^nd^line irinotecan-based systemic chemothera-py after disease progression
Patient 3	yes	no	no	Systemic irinotecan-based chemotherapy with response
Patient 4	yes	yes	no	Systemic irinotecan-based chemotherapy with response
Patient 5	yes	no	no	Systemic irinotecan-based chemotherapy with response
Patient 6	yes	no	no	Systemic irinotecan-based chemotherapy with response
Patient 7	yes	no	no	Systemic irinotecan-based chemotherapy with response
Patient 8	yes	yes	no	Oxaliplatin-associated peripheral neuropathy
Patient 9	yes	no	no	Systemic irinotecan-based chemotherapy with response
Patient 10	yes	yes	no	Progressive disease under oxaliplatin-based systemic chemotherapy
Patient 11	yes	yes	yes	Progressive disease under oxaliplatin-based systemic chemotherapy
Patient 12	yes	no	no	Systemic irinotecan-based chemotherapy with response

CTx = chemotherapy; PM = peritoneal metastasis.

All patients included in the present retrospective study at least completed a 3-year follow-up period. The median follow-up time including events of death was 37.8 months (range 7-51).

Morbidity and toxicity were classified using the Common Terminology Criteria for Adverse Events version 4.0 (CTCAE v4.02) of the U.S. National Cancer Institute. Perioperative mortality was defined as death within 30 days after surgery or in-hospital mortality in case of hospital stay longer than 30 days.

### Cytoreductive surgery

Cytoreductive surgery consists of numerous surgical and peritonectomy procedures depending on the extent of peritoneal tumor dissemination that was determined by the intraoperative calculation of the Peritoneal Cancer Index (PCI) [20, 21]. Operating procedures were performed as described previously [22, 23]. After complete macroscopic cytoreduction (CC-0/1) one inflow drainage, three outflow drainages and two temperature probes were placed in the abdomen to allow the application of HIPEC.

### Hyperthermic intraperitoneal chemotherapy

In all patients bidirectional HIPEC with additional intravenous application of 5-FU at a concentration of 400 mg/sqm body surface and folinic acid at a concentration of 20 mg/sqm body surface about 30 minutes prior to peritoneal chemoperfusion was performed in closed abdomen technique. Abdominal perfusion was started with a total volume of 3 l sodium chloride 0.9% over the inflow drainage using a roller pump system with heat exchanger (ThermaSolutions Inc., Netherlands). Cytostatic agents were added after the temperature in Douglas pouch reached at least 40°C and perfusion was continued for 30 minutes keeping an intraperitoneal temperature of 41-43°C. In the OX group the abdominal cavity was perfused with oxaliplatin at a concentration of 300 mg/sqm body surface and in the IRI group with 300 mg/sqm body surface irinotecan for 30 minutes, respectively.

### Statistics

Kaplan-Maier survival analysis was performed. P-values were calculated using T-test, Chi square and Log rank test as applicable. A two-sided p-value <0.05 was defined to be statistically significant. Statistical analysis was performed using IBM SPSS Statistics for Windows version 19 (SPSS Inc., IBM Corporation, USA).

## Results

### Patients’ characteristics

The mean age of patients was 54 years (range 20-68) with 53 years (range 20-68) in the OX group and 54 years (range 42-66) in the IRI group. Fourteen patients were female and eighteen male. The distribution of American Society of Anaesthesiologists (ASA) scores was 9.4% for ASA I, 71.9% for ASA II and 18.8% for ASA III, respectively.

Patient and tumor characteristics including localization of the primary tumor are summarized in Table 2. The OX group includes a higher number of patients with peritoneal metastasis from appendiceal adenocarcinoma (p = 0.139) and a lower number with primary colon carcinoma (p = 0.073) compared to the IRI group. Nevertheless, the differences were not statistically significant. Moreover, there was a not significant higher number of lymph node negative primary tumors (p = 0.066) and well differentiated adenocarcinomas (G1, p = 0.130) in the OX group. In one patient in the IRI group the T and N status of the primary tumor was not documented.

**Table 2:** **Patient and tumor characteristics**

	Overall	OX	IRI	***p-value***
Number of patients [n]	32	20	12	
I. Patient characteristics				
Mean age (range) [y]	54 (20-68)	53 (20-68)	54 (42-66)	*0.842*
Sex [n] - male	14 (43.8%)	9 (45.0%)	5 (41.7%)	
- female	18 (56.3%)	11 (55.0%)	7 (52.8%)	*1.000*
ASA score [n]				
- ASA I	3 (9.4%)	2 (10.0%)	1 (8.3%)	*1.000*
- ASA II	23 (71.9%)	14 (70.0%)	9 (75.0%)	*1.000*
- ASA III	6 (18.8%)	4 (20.0%)	2 (16.7%)	*1.000*
II. Localization of primary tumor				
Appendix	11 (34.4%)	9 (45.0%)	2 (16.7%)	*0.139*
Colon	7 (21.9%)	2 (10.0%)	5 (41.7%)	*0.073*
Sigma	10 (31.3%)	7 (35.0%)	3 (25.0%)	*0.702*
Rectum	4 (12.5%)	2 (10.0%)	2 (16.7%)	*0.620*
III. Initial TNM classification				
T2	1^*^ (3.2%)	1 (5.0%)	0^**^	*1.000*
T3	10^*^ (32.3%)	6 (30.0%)	4^**^ (36.4%)	*1.000*
T4	20^*^ (64.5%)	13 (65.0%)	7^**^ (63.7%)	*1.000*
N0	16^*^ (51.6%)	13 (65.0%)	3^**^ (27.3%)	*0.066*
N1	5^*^ (16.1%)	2 (10.0%)	3^**^ (27.3%)	*0.317*
N2	10^*^ (32.3%)	5 (25.0%)	5^**^ (45.5%)	*0.423*
M1	22 (68.8%)	14 (70.0%)	8 (66.7%)	*1.000*
G1	5 (15.6%)	5 (25.0%)	0	*0.130*
G2	11 (34.4%)	7 (35.0%)	4 (33.3%)	*1.000*
G3	16 (50.0%)	8 (40.0%)	8 (66.7%)	*0.273*
IV. Further tumor characteristics				
Mean PCI (range)	13 (2-28)	13 (4-28)	12 (2-28)	*0.630*
Synchronous PM	16 (50.0%)	10 (50.0%)	6 (50.0%)	*1.000*
Metachronous PM	10 (31.3%)	6 (30.0%)	4 (33.3%)	*1.000*
Recurrent disease	6 (18.8%)	4 (20.0%)	2 (16.7%)	*1.000*
Liver metastasis	4 (12.5%)	1 (5.0%)	3 (25.0%)	*0.271*
V. Medical history				
Previous abdominal surgery	14 (43.8%)	9 (45.0%)	5 (41.7%)	*1.000*
Previous oncologic surgery	24 (75.0%)	14 (70.0%)	10 (83.3%)	*0.676*
Previous CRS and HIPEC	5 (15.6%)	3 (15.0%)	2 (16.7%)	*1.000*
Previous chemotherapy	28 (87.5%)	16 (80.0%)	12 (100%)	*0.271*
Previous systemic oxaliplatin	17 (53.1%)	11 (55.0%)	6 (50%)	*1.000*

^*^n = 31, ^**^n = 11.

The mean PCI was 13 (range 2-18). 50% of the patients had synchronous and 31.3% metachronous peritoneal metastasis. Six patients showed recurrent disease (18.8%) and four patients had liver metastases at the time of surgery (12.5%). Fourteen patients had previous abdominal surgery (43.8%) and 24 patients (75.0%) already underwent oncologic abdominal surgery for primary tumor or metastasis. Five patients had previous CRS and HIPEC (15.6%). There were no significant differences between the two groups.

Most patients (87.5%) already received systemic chemotherapy during the course of their disease consisting of different chemotherapy regimens. More than half of the patients (53.1%) had previous oxaliplatin-based systemic chemotherapy.

### Operative and perioperative data

Operative and perioperative data is summarized in Table 3. The mean operating time was 348 minutes (range 149-586) with 337 minutes (range 149-586) in the OX group and 366 minutes (range 200-557) in the IRI group, respectively (p = 0.497). The mean blood loss was 271 ml (range 100-600), and the mean number of anastomoses was 1.19 (range 0-3). There were no significant differences between the two groups.

**Table 3:** **Operative and perioperative data**

	Overall	OX	IRI	***p-value***
Number of patients [n]	32	20	12	
I. Operative data				
Mean operating time [min]	348 (149-586)	337	366	*0.497*
Mean blood loss [ml]	271 (100-600)	257	280	*0.760*
Mean no. of anastomoses	1.19	1.21	1.17	*0.899*
II. Perioperative data				
Median stay on ICU [d]	1 (0-6)	1 (0-5)	1.5 (0-6)	*0.332*
Median hospital stay [d]	15.5 (9-42)	15 (9-38)	15.5 (8-42)	*0.863*
Morbidity °3/4 [n]	11 (34.4%)	7 (35.0%)	4 (33.3%)	*1.000*
In-hospital mortality [n]	0	0	0	*1.000*
30-day mortality [n]	0	0	0	*1.000*
Revision surgery [n]	3 (9.4%)	1 (5.0%)	2 (16.7%)	*0.540*

The detailed surgical and peritonectomy procedures are summarized in Table 4. There were no statistically significant differences regarding surgery between the two groups.

**Table 4:** **Peritonectomy and surgical procedures**

	Overall	OX	IRI	***p-value***
Number of patients [n]	32	20	12	
Greater omentectomy	24	17	7	*0.204*
Upper right peritonectomy	21	14	7	*0.703*
Upper left peritonectomy	7	5	2	*0.683*
Parietal peritonectomy	9	6	3	*1.000*
Pelvic peritonectomy	22	15	7	*0.438*
Small bowel resection	19	12	7	*1.000*
Colonic resection	17	11	6	*1.000*
Rectal resection	20	13	7	*0.999*
Cholecystectomy	9	6	3	*1.000*
Liver resection	5	1	4	*0.053*
Gastric resection	3	1	2	*0.540*
Splenectomy	6	3	3	*0.647*
Ovarectomy	2	0	2	*0.133*
Hysterectomy	3	1	2	*0.540*
Vesical resection	2	1	1	*1.000*
Loop ileostomy	4	3	1	*1.000*
Overall number of peritonectomy procedures	83	57	26	*0.104*
Overall number of visceral resections	86	49	37	*0.142*

The median hospital stay was 15 days (range 9-38) in the OX group and 15.5 days (range 8-42) in the IRI group, respectively (p = 0.863). The median stay on ICU was one day (range 0-6) in both groups (p = 0.332).

### Morbidity and mortality

The overall grade 3/4 morbidity rate according to CTCAE v4.02 was 34.4% with 35.0% in the OX group and 33.3% in the IRI group, respectively (p = 1.000). Postoperative complications are summarized in detail in Table 5. In the OX group two patients developed pleural effusion requiring intervention. Moreover, ileus, intraabdominal abscess, bowel perforation, lung embolism, and cardiac arrhythmia were observed. In the IRI group ileus, postoperative bleeding, wound infection and pneumonia occurred. There was no documented hematological toxicity requiring intervention in both groups. Three patients (9.4%), one patient in the OX group (5.0%) and two patients in the IRI group (16.7%), required revision surgery due to postoperative complications (p = 0.540). Reasons for re-operation were postoperative bleeding, small bowel perforation and extensive wound infection.

**Table 5:** **Postoperative complications grade 3/4**

	Overall	OX	IRI	***p-value***
Number of patients [n]	32	20	12	
Pleural effusion	2 (6.3%)	2 (10.0%)	0	*0.516*
Pneumonia	1 (3.1%)	0	1 (8.3%)	*0.375*
Lung embolism	1 (3.1%)	1 (5.0%)	0	*1.000*
Bowel perforation	1 (3.1%)	1 (5.0%)	0	*1.000*
Ileus	2 (6.3%)	1 (5.0%)	1 (8.3%)	*1.000*
Postoperative bleeding	1 (3.1%)	0	1 (8.3%)	*0.375*
Wound infection	1 (3.1%)	0	1 (8.3%)	*0.375*
Intraabdominal abscess	1 (3.1%)	1 (5.0%)	0	*1.000*
Cardiac arrhythmia	1 (3.1%)	1 (5.0%)	0	*1.000*

Perioperative morbidity defined as 30-day or in-hospital mortality (depending on the length of hospital stay) was 0% in both groups.

### Survival analysis

**Figure 1:**
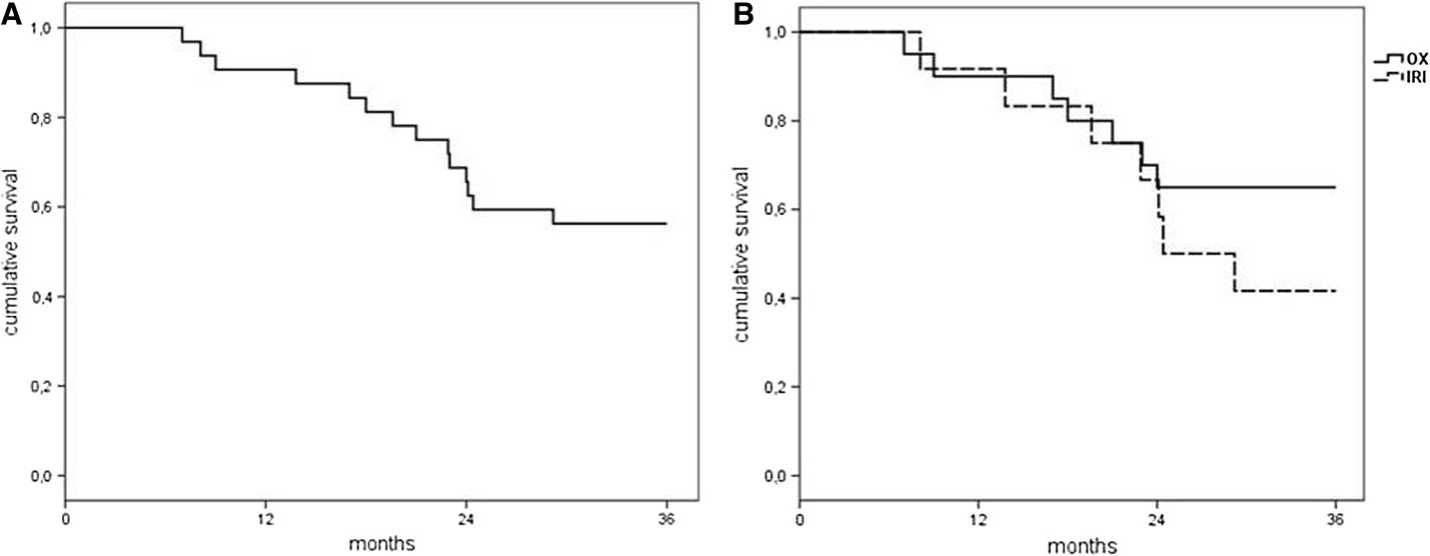
**Kaplan-Meier survival analysis. A** - Overall survival. n =32. 2-year survival rate 68.8%, 3-year survival rate 56.3%. **B** - group-specific overall survival. 2-year survival rates: 70.0% (OX) and 66.7% (IRI), 3-year survival rates: 65.0% (OX) and 41.7% (IRI). p =0.295 (n.s.). OX = oxaliplatin-based HIPEC, IRI = irinotecan-based HIPEC.

**Figure 2:**
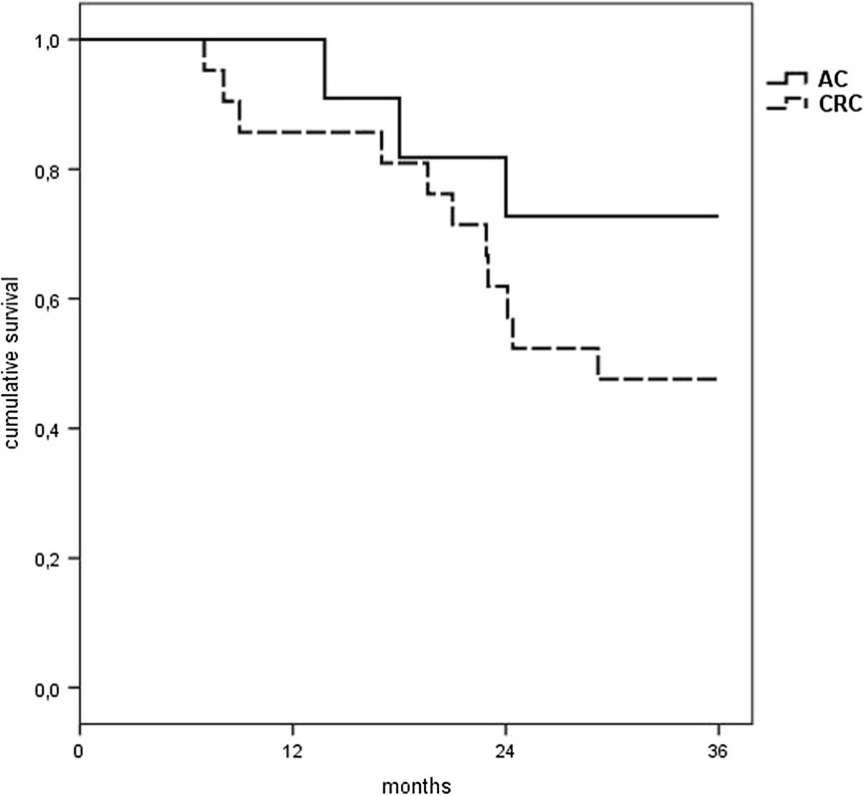
**3-year survival depending on primary tumor.** Overall survival of patients with appendiceal cancer (n = 11) vs. patients with colorectal cancer (n = 21) as primary tumor. 3-year survival rates: 72.7% (AC) and 47.6% (CRC). p = 0.213 (n.s.). AC = appendiceal cancer, CRC =  colorectal cancer.

The overall 2-year and 3-year survival rates were 68.8% and 56.3%, respectively (Figure 1A). The group-specific 2-year survival rates were 70.0% in the OX group and 66.7% in the IRI group (p = 0.846). The 3-year survival rates reached 65.0% in the OX group and 41.7% in the IRI group (p = 0.295). This difference was not statistically significant (Figure 1B). In the OX group median survival has not yet been reached during follow-up. The median follow-up time including events of death in this group of patients was 39.4 months (range 7.2-51.1). The IRI group showed a median survival of 26.8 months (95% CI 15.7-33.1 months).Subgroup analysis showed a 3-year overall survival rate of 72.7% in 11 patients with appendiceal primary compared to 47.6% in 21 patients with peritoneal metastasis arising from colonic, sigmoid or rectal cancer (Figure 2, p = 0.213). After three years 68.8% of patients with negative initial lymph node status survived (n = 16) compared to 46.7% of patients with positive lymph nodes (n = 15) at time of first diagnosis (p = 0.231). There was also no statistically significant difference regarding 3-year survival rates depending on the histological grading. Overall 3-year survival rates were 60.0% for patients with well differentiated primary tumors (G1, n = 5), 54.5% for patients with moderately differentiated primary tumors (G2, n = 11) and 53.6% for patients with poorly differentiated primary tumors (G3, n = 16), respectively (p =0.998).

## Discussion

The multimodality treatment concept of cytoreductive surgery (CRS) and hyperthermic intraperitoneal chemotherapy (HIPEC) is associated with significant rates perioperative morbidity. Beyond patient factors such as comorbidities the operative risk of CRS depends on the extent of surgery and the distinctive features of the performed surgical procedures. HIPEC may cause additional chemotherapy-related complications and toxicity. In the literature morbidity rates in specialized peritoneal carcinomatosis centers range from 23% to 45% depending on the assessment and definition of perioperative complications [3, 4, 7, 24–27]. Quenet et al. reported an in increase of the morbidity rate from 34.9% to 52.4% by adding intraperitoneal irinotecan to an oxaliplatin-based bidirectional HIPEC regimen [12]. In the present study the overall grade 3/4 morbidity rate was 34.4%. Although pharmacokinetic studies on heated intraperitoneal oxaliplatin reported dose absorption rates from 40% to 68% within 30 minutes perfusion [28, 29], we found no hematologic toxicity requiring intervention neither in the OX nor in the IRI group. This result is consistent with previous observations [30, 31]. Nevertheless, Elias et al. reported a haematological toxicity rate of 11% after bidirectional HIPEC with intraperitoneal oxaliplatin plus irinotecan [18]. In a systematic review including numerous different HIPEC regimens the mean overall rate of hematologic toxicity was 5.6% [32]. The low chemotherapy-related morbidity in our series may be caused by the concentration of 300 mg/sqm body surface in comparison to 460 mg/sqm body surface in the French series. In a recently published study of oxaliplatin pharmacokinetics during bidirectional HIPEC the oxaliplatin dose has been reduced from 460 mg/sqm body surface to 360 mg/sqm body surface after the first 17 patients due to toxicity [33].

In the present series three patients had to be re-operated for perioperative complications (9.4%). In the literature revision surgery is reported for 8.2% to 14% of patients that underwent CRS and HIPEC [3, 25, 26, 34]. The recently published data from the American College of Surgeons National Surgical Quality Improvement Program showed a re-operation rate of 10% [35]. There was no perioperative mortality in the present study. In a systematic review of 155 articles published by Chua et al. the mean mortality rate was 2.9% ranging from 0% to 17% [32]. In the American College of Surgeons National Surgical Quality Improvement Program hospitals overall morbidity rate was 2% [35]. Hospital stay and stay on ICU did not differ between the two groups in our series and were comparable to published data [3, 7, 9, 24, 30, 34, 35].

The safety and efficacy of intravenous oxaliplatin and irinotecan in combination with 5-FU and folinic acid has been demonstrated in numerous studies [36–43]. Both cytostatic agents are considered to be part of the standard treatment of advanced colorectal cancer. Nevertheless, systemic treatment is less efficient in patients with peritoneal metastasis [44]. Franko et al. compared the outcome of 2,095 patients enrolled onto two prospective randomized clinical trials evaluating systemic chemotherapy for patients with CRC with (pcCRC) and without peritoneal metastasis (non-pcCRC) and reported median survival of 12.7 months in the pcCRC group (n = 364) vs. 17.6 months in the non-pcCRC group. In this analysis infusional oxaliplatin-based chemotherapy was superior to irinotecan in first line therapy of pcCRC patients [14].

Based on the successful use of systemic oxaliplatin, 5-fluorouracil and folinic acid in patients with mCRC bidirectional HIPEC with intraperitoneal oxaliplatin and intravenous 5-FU/folinic acid has been used within the multimodality concept of CRS and HIPEC in patients with peritoneal metastasis arising from CRC. Elias et al. reported promising results with a median survival of 62.7 months and a 5-year survival rate of 51% [11]. In a prospective phase II study published by Hompes et al. the 2-year overall survival rate reached 88.7% and the median disease-free survival (DFS) was 19.8 months [45]. Consistent with this data the 3-year survival rate in our series was 65.0%. In contrast to the results for systemic treatment using the FOLFOXIRI protocol published by Falcone et al. [38] addition of intraperitoneal irinotecan to the bidirectional oxaliplatin-based HIPEC regimen did not lead to improved overall or relapse-free survival. Quenet et al. reported a median overall survival 47 months and a 5-year survival rate of 42.4% [12]. The survival data of a phase I trial combining irinotecan with mitomycin C for HIPEC is not yet available [19].

In the present retrospective analysis the rationale for intraperitoneal irinotecan was response to systemic irinotecan-based chemotherapy in seven patients, disease progression under oxaliplatin-based systemic chemotherapy in three and oxaliplatin-associated peripheral polyneuropathy ≥ grade 3 in two patients. Median overall survival was 26.8 months and 3-year survival rate 41.7% in the IRI group suggesting a negative trend compared to the OX group as well as most published survival data after complete macroscopic cytoreduction and HIPEC with other cytostatic agents or combinations [8, 9, 11, 45]. Nevertheless, in a recently published retrospective analysis of 95 patients Hompes et al. reported a median overall survival of 37.1 months after oxaliplatin-based HIPEC and 26.5 months for the mitomycin C-based HIPEC after complete macroscopic cytoreduction [46]. This observation is supported by the data published from the American Society of Peritoneal Surface Malignancies showing a median overall survival of 32.7 months in patients treated with complete macroscopic cytoreduction and MMC-based HIPEC vs. 31.4 months in patients after CC-0/1-resection and oxaliplatin-based HIPEC [47]. Due to the small number of patients in the present series the survival difference between the two groups was not statistical significant (p = 0.295) and therefore the relevance of this observation is limited. Moreover, there were a statistically not significant lower number of patients with appendiceal cancer and G1 differentiation as well as more patients with positive lymph node status in the IRI group. In a retrospective study Elias et al. reported a statistically significant improved overall 5-year survival rate of 63% for patients with peritoneal metastasis arising from appendiceal adenocarcinoma in comparison to colon (29.7%) and rectal cancer (37.9%). In the multivariate analysis positive lymph node status was an independent negative prognostic factor (p = 0.001) [48]. This observation has been confirmed by other published data [49, 50]. Nevertheless, Jimenez et al. reported a median survival of 47 months and a 5-year survival rate of 41% after CRS and HIPEC in 125 patients with histologically proven peritoneal carcinomatosis (PMCA) arising from appendiceal cancer [51]. This survival data is comparable to the survival rates published for patients with colorectal peritoneal metastasis. However, in the present series the subgroup analysis of tumor origin, grading and lymph node status showed no significant differences.

## Conclusions

In conclusion, our data show that both bidirectional HIPEC regimens may be used with comparable low mortality and acceptable morbidity in a specialized peritoneal carcinomatosis center. Published data and the positive trend regarding the overall 3-year survival rate in the present series support oxaliplatin-based HIPEC as the first choice treatment regimen. Nevertheless, in our opinion irinotecan-based HIPEC should still be considered as a promising alternative in patients with tumor progression or intolerable toxicity under chemotherapy with oxaliplatin. However, comparative prospective randomized trials are necessary to determine the best treatment regimen regarding morbidity, mortality and particularly long-term oncological outcome.

## Consent

Written informed consent was obtained from the patient for inclusion into the database and publication of the data.

## Abbreviations

5-FU: Fluorouracil
ASA: American Society of Anesthesiologists
CA19-9: Carbohydrate antigen 19-9
CC: Completeness of cytoreduction
CEA: Carcinoembryonic antigen
CI: Confidence interval
CRC: Colorectal cancer
CRS: Cytoreductive surgery
CT: Computed tomography
CTCAE: Common Terminology Criteria for Adverse Events
DPAM: Disseminated peritoneal adenomucinosis
FA: Folinic acid
FOLFIRI: Folinic acid + fluorouracil + irinotecan
FOLFOX: Folinic acid + fluorouracil + oxaliplatin
FOLFOXIRI: Folinic acid + fluorouracil + irinotecan + oxaliplatin
HIPEC: Hyperthermic intraperitoneal chemotherapy
mCRC: Metastatic colorectal cancer
MMC: Mitomycin C
OS: Overall survival
pcCRC: Peritoneal carcinomatosis of CRC
PCI: Peritoneal Cancer Index
PFS: Progression-free survival
PM: Peritoneal metastases
PMCA-I: Peritoneal mucinous carcinomatosis of intermediate features
QoL: Quality of life
RCT: Randomized controlled trial.
